# Essential Engagement as the First Step in Gaining Entrée into the Laotian American Community on Cervical Cancer Screening

**DOI:** 10.31372/20200503.1095

**Published:** 2020

**Authors:** Catherine Pravisay-Malmstadt, Connie K. Y. Nguyen-Truong

**Affiliations:** a Washington State University Elson S. Floyd College of Medicine in Spokane, United States; b Washington State University College of Nursing in Vancouver, Vancouver, United States; c Dr. Connie Kim Yen Nguyen-Truong is the last senior author

**Keywords:** Laotian American women, Laotian, community, cervical cancer screening, engagement, cultural, community leaders, stakeholders, culturally sensitive interactions

## Introduction

Asian American women, including Laotian American women (LAW), have the lowest rate of being up to date with cervical cancer (CC) screenings at 75% compared to other ethnic groups (85% White, 86% Black, 79% Hispanic, 79% American Indian/Alaska Native; American Cancer Society, 2019; Nghiem, Davies, Chan, Mulla, & Cantor, 2016). This rate is substantially lower than the national objective of 93% (Healthy People.gov, 2020). CC is highly treatable if caught early in the localized stage with a 91.8% 5-year survival rate (National Cancer Institute, n.d.). There is scant research on the incidence and factors surrounding CC screening in Laotian Americans and has primarily been representative of California. The Portland metropolitan area in the United States’ (U.S.) Pacific Northwest has one of the top ten highest Laotian American populations (Greblo, 2011). The Laotian American cultural community leaders (CCLs) in the Pacific Northwest expressed to our academic project team at Washington State University Elson S. Floyd College of Medicine and the College of Nursing that the Laotian American community is a private ethnic group wary of those from the outside and particularly researchers. Research evidence points to the importance of meaningful stakeholder engagement in scholarly work (Bourassa et al., 2020; Dill et al., 2020; Hoekstra et al., 2020; ^1^Nguyen-Truong, ^1^Fritz et al., 2018; Nguyen-Truong, Tang, & Hsiao, 2017; Wallenstein, Duran, Oetzel, & Minkler, 2018). The purpose of this brief article is to describe the first essential engagement step of relationship building between the academic project team and Laotian American CCLs to gain entrée to the Laotian American community regarding a sensitive topic on gynecologic health.

## Problem/Significance of Topic

Some LAW in the Pacific Northwest community are CC survivors and expressed a need to learn about CC screening among LAW, given the high curability and survival rates if caught early. Earlier research from Dang, Lee, and Tran (2010) suggests that CC screening is not often discussed among LAW. Only 62.3% of LAW reported having family or friends who encouraged them to get CC screening rather than from their healthcare provider or health education sources (Dang et al., 2010). Therefore, a foundational engagement step in collaborating with Laotian American CCLs is essential to gain entrée into the Laotian American community for CC screening.

It is important to first gain trust and build rapport with CCLs. In a review, Hoekstra et al. (2020) found ethical issues regarding collaborative activities, including needing additional time, financial costs, uncomfortable feelings related to sharing power, and disempowerment among minoritized ethnic groups. Thus, meaningful cultural immersion experiences for students, colearning, and scholarly work practices are crucial for relationship building and practice (Thackrah, Hall, Fitzgerald, & Thompson, 2017). Dill et al. (2020) found that engaging with community stakeholders early in the project design planning ensures their voices are amplified in the process. Academic and community stakeholders’ interactions may impact the engagement, colearning, project continuity, and cultural immersion experience. Building an authentic relationship requires ethically serving the community and paying particular attention to ensure success of the interactions (Bourassa et al., 2020).

## Methods

The project team consists of a Medical Doctor (MD) Candidate who is Laotian American and a Vietnamese American academic nurse scientist who has a background in qualitative methodology, cancer control, and early detection, and communities of color including immigrants and refugees. The academic nurse scientist is also a community leader and has a trusted partnership with an immigrant and refugee-based community organization in the U.S.’ Pacific Northwest (Immigrant & Refugee Community Organization, 2001–2020). The Washington State University Office of Research Assurances has certified exemption (17824-001) for this project. The project team drew on research evidence regarding leveraging strengths from academic teams and CCLs and culturally sensitive interactions with Asian communities as a guide to the essential engagement as the first step in gaining entrée to the Laotian American community (Bourassa et al., 2020; Dill et al., 2020; Hoekstra et al., 2020; ^1^Nguyen-Truong, ^1^Fritz et al., 2018; Nguyen-Truong et al., 2017; Wallenstein et al., 2018). The essential engagement step included trust and rapport building, conveying respectful and authentic intentions on a shared mission of learning about CC screening in LAW, and time needed for building connections and multiple conversations with CCLs in the Laotian American community.

The project team met 19 times with four Laotian American CCLs who are known and respected elders in the community. CCLs included the elder liaison, the President, and the Treasurer at the Lao Women Association community-based organization, and the Representative of the LAW elders. The meetings were held by phone and videoconferencing due to geographic distance and social distancing efforts during the COVID-19 pandemic. The meetings each lasted about one hour. The meetings focused on getting to know one another; what the main project means for the community; and mutual agreements such as the project purpose, outreach, recruitment, interview methods, and sharing findings with the community. The project team used the Plus/Delta method to evaluate the strengths and whether improvements might be necessary regarding the engagement process (O’Connell & Vandas, 2015). The project team obtained verbal consent to document. The MD Candidate documented field notes-based data via a journal that included the observations of the interactions, views, and thought process with CCLs and verified through discussions.

## Results

The project team identified three main themes.

The first theme regards Laotian American elders helping to bridge the project team from academia to the Lao Women Association. The project team was able to obtain commitment from a trusted and respected elder as a liaison who reported appreciation for caring about the Laotian American community and working with CCLs. The elder liaison endorsed the importance of collaborating to benefit the Laotian American community. The elder helped to bridge authentic conversations with cultural sensitivity between the project team and a trusted and respected President at the Lao Women Association. For example, these bridging efforts included learning to speak in a hierarchical manner and emphasizing respect and humility when speaking to elders such as referring to elders as “uncle”/ (Laotian translation) or “aunt”/ (Laotian translation). The President helped to bridge authentic conversations to the Treasurer and the Representative of LAW elders.

The second theme is having arrived on a shared mission to respond to a need identified by the Laotian American community. For example, the Laotian American President demonstrated passion and enthusiasm and was in support of describing perceptions and understanding of CC screening. CCLs were excited for the opportunity to be a part of the “research world.” Endorsement from the Laotian American President gave credibility to both the main project and the project team through word of mouth throughout the Laotian American community.

The third theme is humanizing the connections through stories. For example, the first meeting was framed around humanizing the connections through an exchange of stories regarding familial background between the MD Candidate and the CCLs as a cultural custom. CCLs expressed that this sensitivity to cultural customs helped with feeling comfortable working with the project team.

## Discussion

The essential engagement step worked as planned. The elders who were helping bridge the project team to the other CCLs at the Lao Women Association underscored the importance of authentic conversations regarding collaboration. The culturally sensitive communication interactions and mannerisms between the project team and CCLs demonstrated respect for the Laotian American elders and community. The trust and rapport building that occurred with the project team and CCLs at the beginning of the project on a shared mission allowed CCLs to feel comfortable reaching out to the team throughout the project for updates and vice versa. The investment from everyone involved included valuable collaboration time and substantive effort, open communication, and using an academic and Laotian cultural lens. The numerous meetings and interactions with CCLs allowed the necessary time for sharing stories. The MD Candidate had the needed time to become trusted as a person and as the project team. The project team was able to successfully engage with the CCLs and gained entrée into the Laotian American community.

## Recommendations

Researchers and community stakeholders would mutually benefit from authentic engagement from the beginning of the project and remaining engaged throughout the project implementation. Balancing regular and consistent culturally sensitive interactions with CCLs can add richness to the project purpose. Respecting a private ethnic community who self-identify as not accustomed to being involved in research is necessary. Researchers need to allocate the appropriate time for trust and rapport building.

## Acknowledgments

The authors thank Dr. Anjali Kumar, MD, MPH, FACS, FASCRS, Director, Clinical Education–Surgery at Washington State University (WSU) Elson S. Floyd College of Medicine who provided a review of the main project. The authors thank the Lao Women Association for their support of the project. The authors are appreciative of Dr. Jack Downs, PhD, at WSU Health Sciences Spokane Student Success Center and Sharalee Chwaliszewski at WSU Vancouver Writing Center for editing assistance on an earlier version. The authors are also appreciative of the anonymous peer reviewers for assistance.

## Funding

The authors are appreciative of funding that supported in part the project. Catherine Pravisay-Malmstadt, BS, was awarded the Washington State University Elson S. Floyd College of Medicine Student Scholarship with Dr. Connie Kim Yen Nguyen-Truong, PhD, RN, Alumnus PCCN as the principal investigator project mentor.
